# Sexual Dimorphism of Early Transcriptional Reprogramming in Dorsal Root Ganglia After Peripheral Nerve Injury

**DOI:** 10.3389/fnmol.2021.779024

**Published:** 2021-12-13

**Authors:** Andrei V. Chernov, Veronica I. Shubayev

**Affiliations:** ^1^Department of Anesthesiology, University of California, San Diego, San Diego, CA, United States; ^2^VA San Diego Healthcare System, San Diego, CA, United States

**Keywords:** Mus musculus, sexual dimorphism, peripheral nerve injury, axotomy, RNA-seq, dorsal root ganglia

## Abstract

Peripheral nerve injury induces genome-wide transcriptional reprogramming of first-order neurons and auxiliary cells of dorsal root ganglia (DRG). Accumulating experimental evidence suggests that onset and mechanistic principles of post-nerve injury processes are sexually dimorphic. We examined largely understudied aspects of early transcriptional events in DRG within 24 h after sciatic nerve axotomy in mice of both sexes. Using high-depth RNA sequencing (>50 million reads/sample) to pinpoint sexually dimorphic changes related to regeneration, immune response, bioenergy, and sensory functions, we identified a higher number of transcriptional changes in male relative to female DRG. In males, the decline in ion channel transcripts was accompanied by the induction of innate immune cascades *via* TLR, chemokine, and Csf1-receptor axis and robust regenerative programs driven by Sox, Twist1/2, and Pax5/9 transcription factors. Females demonstrated nerve injury-specific transcriptional co-activation of the actinin 2 network. The predicted upstream regulators and interactive networks highlighted the role of novel epigenetic factors and genetic linkage to sex chromosomes as hallmarks of gene regulation post-axotomy. We implicated epigenetic X chromosome inactivation in the regulation of immune response activity uniquely in females. Sexually dimorphic regulation of MMP/ADAMTS metalloproteinases and their intrinsic X-linked regulator Timp1 contributes to extracellular matrix remodeling integrated with pro-regenerative and immune functions. *Lexis1* non-coding RNA involved in LXR-mediated lipid metabolism was identified as a novel nerve injury marker. Together, our data identified unique early response triggers of sex-specific peripheral nerve injury regulation to gain mechanistic insights into the origin of female- and male-prevalent sensory neuropathies.

## Introduction

Sensory deficits arising after peripheral nerve injury (PNI) or disease can impact touch, vibration, position, temperature, and pain senses. Regeneration of the damaged sensory neurons is a complex process orchestrated in part by transcriptional events at the level of the neuron cell bodies localized in dorsal root ganglia (DRG; Gordon et al., 2003; McDonald et al., 2006; Zochodne, 2012). Each DRG contains thousands of cell bodies of phenotypically distinct primary afferent neurons, each enveloped by a layer of satellite glia and surrounded by resident and infiltrating cells of immune, stromal, endothelial, and other origins (Devor, 1999; Lawson, 2002; Takeda et al., 2009). The diversity of cell populations ensures dynamic DRG transcriptomes in humans (Flegel et al., 2015; LaPaglia et al., 2018; Ray et al., 2018; North et al., 2019). Rodent models of PNI have provided a valuable tool to capture extensive transcriptional reprogramming gradually unfolding at the site of nerve injury and the corresponding DRG and spinal cord tissues (Kubo et al., 2002; Xiao et al., 2002; Bosse et al., 2006; Costigan et al., 2009; Michaelevski et al., 2010; LaCroix-Fralish et al., 2011; Kim et al., 2012; Li et al., 2013; Yao et al., 2013; Jiang et al., 2014; Perkins et al., 2014; Chernov et al., 2015, 2020; Chandran et al., 2016; He and Jin, 2016; Mahar and Cavalli, 2018; Renthal et al., 2020). In addition, the transcriptional makeup of DRG is enriched by axonally trafficked (Devor, 1999; Takeda et al., 2009) and exosome-delivered (Simeoli et al., 2017) coding and non-coding (nc) RNAs in a dynamic manner in the course of PNI response.

Sex is increasingly recognized as a critical biological variable in the functional repair of damaged sensory neurons. Inflammatory, neuropathic, and idiopathic hypersensitivity and persistent pain conditions often have greater prevalence and symptom severity in women as compared to men (Unruh, 1996; Greenspan et al., 2007; Fillingim et al., 2009; Mogil, 2012; Sorge and Totsch, 2017; Boerner et al., 2018). Transcriptome RNA-sequencing (RNA-seq)-based analyses of human DRG at baseline (Ray et al., 2019) and in patients with radicular/neuropathic pain (North et al., 2019) highlighted DRG’s central role in sex-specific gene regulation. Likewise, emerging research has revealed sexual dimorphism in rodent DRG transcriptomes in response to peripheral nerve injury (Stephens et al., 2019; Ahlström et al., 2021), hyperalgesic priming by interleukin 6 (Il-6; Paige et al., 2020), sciatic nerve injection of myelin basic protein (MBP) derived peptides (Chernov et al., 2020), and other stimuli. Transcriptome differences in DRG have been implicated in female-prevalent hypersensitivity partly due to the activity of prostaglandin signaling and neuroendocrine mechanisms involving prolactin receptors (North et al., 2019; Ray et al., 2019; Chernov et al., 2020; Mecklenburg et al., 2020; Paige et al., 2020; Tavares-Ferreira et al., 2020).

Rapid initiation of regeneration is essential to the successful functional repair of sensory neurons in PNI (Gordon et al., 2003; McDonald et al., 2006; Zochodne, 2012). Within 24 h after sciatic nerve transection in rodents, DRG transcriptome induces neuron-specific regeneration associated genes (RAGs), including activating transcription factor 3 (*Atf3*), and growth-associated protein (*Gap)43* (Bolin et al., 1995; Tsujino et al., 2000; Bosse et al., 2001; Snider et al., 2002; Sun and He, 2010; Van Kesteren et al., 2011; Ma and Willis, 2015; Chandran et al., 2016; Rao and Pearse, 2016; Renthal et al., 2020; Shiers et al., 2020; Suzuki and Ochi, 2020; Lee and Cho, 2021). Shortly after peripheral nerve injury, the expression of genes encoding cytokines, chemokines, Toll-like receptors (TLRs), matrix metalloproteinases (MMPs), and other mechanistically related proteins increased (Kim et al., 2012; Chernov et al., 2015). Both resident and infiltrating cell types in DRG can promote sex-specific responses to nerve injury (Mapplebeck et al., 2016, 2017). The injured sensory neurons engage molecular crosstalk with DRG macrophages *via* colony-stimulating factor 1 (Csf1) to stimulate male-specific macrophage expansion at day four post-PNI (spared nerve injury) (Yu et al., 2020). As a result, these processes contribute to the initiation and maintenance of hypersensitivity in a sex-specific manner.

It remains unknown which early-response DRG transcriptome changes display sexual dimorphism in response to PNI. Using high-depth RNA-seq, the present study has identified significant sex-specific early-response genome-wide transcriptional changes in coding and ncRNA in male and female DRG at 24 h after sciatic nerve axotomy in mice. Applying predictive bioinformatics, we determined signaling pathways, particularly those related to regenerative, immune, metabolic, sex hormone, and X/Y chromosome-linked systems.

## Results

To identify sexually dimorphic early-response PNI-specific molecular signatures, we conducted sciatic nerve complete axotomy (Anderson, 1902; Ramon y Cajal, 1928) in female and male mice according to our established procedure (Chattopadhyay and Shubayev, 2009; Chernov et al., 2015). At 24 h after sham or axotomy surgery (*n* = 6/group; pooled *n* = 2/sample), ipsilateral lumbar (L)4 and L5 (pooled) DRG were processed using RNA-seq. Genome-wide gene regulation and gene ontology analyses were performed as specified below.

### Early-Response Genome-Wide Transcriptome in Dorsal Root Ganglia After Peripheral Nerve Injury

A total of 140,789 individual transcripts were identified in DRG across four groups, corresponding to 54,309 annotated genes. Following exclusion of genes with low abundance measurements [count per million (CPM) <10 across four groups], 26,518 genes were analyzed for differential regulation between axotomy and sham groups in female and male DRG.

**FIGURE 1:**
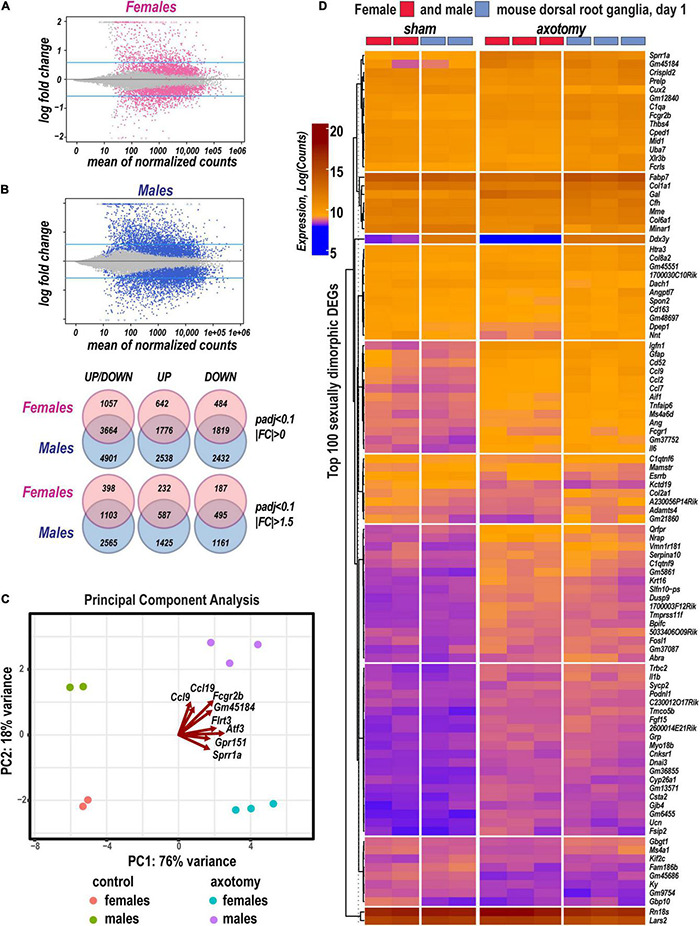
Sexually dimorphic transcriptional changes in murine female and male DRG 24 h after axotomy. **(A)** Bland-Altman plots of shrunken log2FC and log2 of Mean Average counts (MAplot function in the DESeq2 suit). Adjusted log2FC were calculated using the adaptive *t*-prior *apeglm* method (lfcShrink function in DESeq2 suit). Female and male DEGs with padj < 0.1 are marked as red or blue points, respectively. Points are colored gray if padj > 0.1. Points that fall out of the displayed window are plotted as open triangles pointing either up or down. Blue horizontal lines indicate Log2FC > 0.58 and Log2FC < −0.58 thresholds. **(B)** Venn diagrams (left to right) demonstrate a number of total identified DEGs (padj < 0.1, log2FC≠0), upregulated DEGs [padj < 0.1, Log2FC > 0 (top graph) or Log2FC > 0.58 (bottom graphs)], or downregulated DEGs [padj < 0.1, Log2FC < 0 (top graph) or Log2FC < −0.58 (bottom graphs)] in female (red circles) and male (blue circles) mice. The intersections of circles correspond to unidirectionally changed DEGs. **(C)** Principal component (PC) analysis of DEGs in three replicate groups of female and male DRG and the respective sham-operated controls. Blue and red indicate males and females, respectively. Red arrows indicate genes with the most significant weight. **(D)** Hierarchical clusters of the most significant up- and downregulated DEGs (padj < 0.1). Heatmap colors correspond to gene abundance (Log counts) following variance stabilizing transformation (DESeq2).

DESeq2 workflow used the Wald test (Love et al., 2014) to quantify significance parameters and predict differentially expressed genes (DEGs; Figure 1A). In addition, an adaptive *t*-prior shrinking (*apeglm)* allowed to rank DEGs by the “effect size” and reduce noise associated with higher fold change (FC) from low abundance genes (Zhu et al., 2019). Over 10,600 significant DEGs with adjusted *p*-values (padj) below a false discovery rate (FDR) cutoff (padj < 0.1) were further analyzed (Supplementary Table 1). We identified 2,418 upregulated and 2,303 downregulated DEGs in females, and 4,314 upregulated and 4,251 downregulated DEGs in males (Figure 1B). A total of 3,664 DEGs exhibited monomorphic regulation in both sexes, including 1,776 and 1,819 up- and downregulated genes, respectively. Of all DEGs, 642 and 2,538 genes were uniquely upregulated in females or males, respectively; 484 and 2,432 genes were uniquely downregulated in females or males, respectively. Over 42% of DEGs exhibited FC above 1.5 or below −1.5.

### Functional Early-Response Programs in Dorsal Root Ganglia After Peripheral Nerve Injury

**FIGURE 2:**
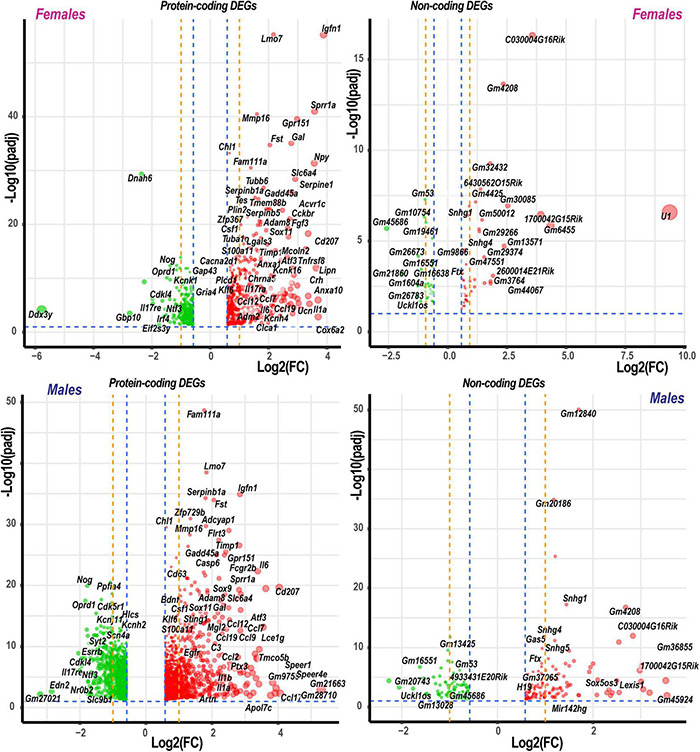
Significant DEGs in females and males. Significance [–log10(padj)] versus log2 fold change (FC) volcano plots of protein-coding and ncRNA DEGs in females and males. Red and green colors indicated up- and downregulated DEGs, respectively. The diameter of each circle is proportional to the respective log2FC. Only data above thresholds (padj < 0.1, | Log2FC| > 0.58, indicated by blue dashed lines) are displayed. Dotted orange lines indicate stringent | Log2FC| > 1 (| FC| > 2) thresholds. Select DEGs are labeled.

**FIGURE 3:**
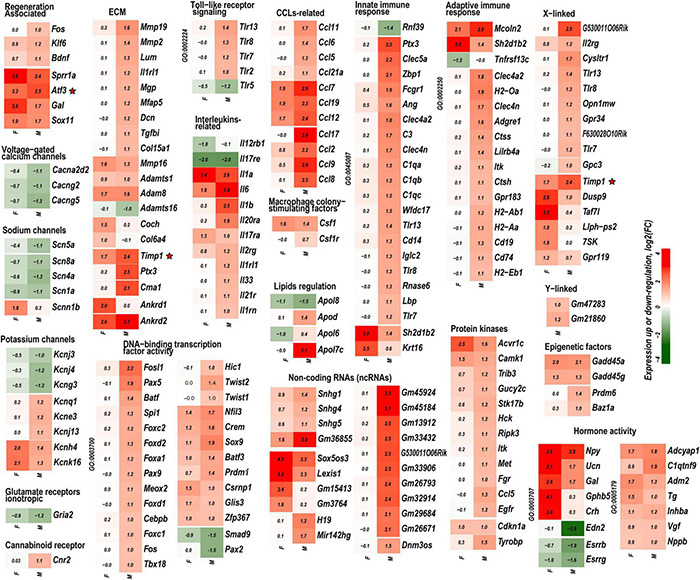
Mono- and dimorphic regulation of select gene families grouped by GO terms, functional similarity, structure, genomic co-localization, and published study datasets. Only DEGs with significance scores (padj < 0.1) and | Log2FC| > 1 (| FC| > 2) are shown. Up- and downregulated DEGs are colored according to the respective color scales of log2FC. GO term identification numbers are shown then applicable on the left side of each heatmap. Red stars indicate genes investigated by protein immunoblotting. A complete list of DEGs is included in Supplementary Table 1.

Based on the principal component analysis (PCA) of 1,000 top-regulated DEGs (Figure 1C), we attributed 76% (PC1) and 18% (PC2) variance to PNI and biological sex, respectively. In PNI, the variance due to sexually dimorphic regulation was higher than in sham. Hierarchical clustering (Figure 1D) highlighted genes related to nerve regeneration, ion channels, transporters, extracellular matrix (ECM), immune, metabolic, and transcriptional regulators. The statistically significant protein-coding and ncRNA DEGs prevailing in each sex are visualized on volcano plots (Figure 2). We conducted in-depth analyses of DEGs within specific gene ontology (GO) categories, gene families with mechanistic relevance to PNI response (Figure 3 and Supplementary Table 1).

#### Nerve Regeneration

**FIGURE 4:**
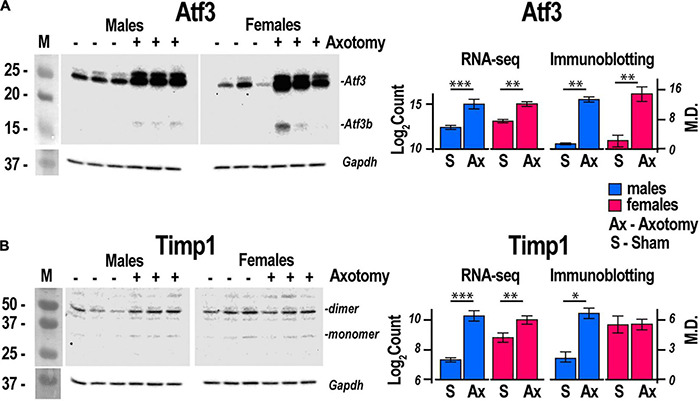
Atf3 and Timp1 protein expression correlated with transcriptional activity of respective genes. Axotomy or sham surgeries were conducted in male and female mice (*n* = 3/group). After 24 h, DRGs ipsilateral to the surgery site were dissected and total proteins were analyzed by immunoblotting. Primary antibodies to mouse **(A)** Atf3 and **(B)** Timp1 were used. Gapdh served as loading control. Right panels, comparison of absolute expression Log_2_Count of **(A)** Atf3, and **(B)** Timp1 with densitometry analysis of protein bands by immunoblotting. Digitized immunoblotting data from three individual replicates were used to calculate means and standard deviation. ANOVA with *post hoc* Tukey test was applied to determine statistical significance: * *p* < 0.01; ** *p* < 0.001; *** *p* < 0.0001. M, molecular weight marker; Log_2_Count, Log_2_ normalized transcript counts; M.D., mean density; S, Sham, Ax, Axotomy.

Regeneration associated genes are neuron-intrinsic factors that stimulate axonal outgrowth after PNI (Bolin et al., 1995; Tsujino et al., 2000; Bosse et al., 2001; Snider et al., 2002; Abe and Cavalli, 2008; Sun and He, 2010; Van Kesteren et al., 2011; Ma and Willis, 2015; Chandran et al., 2016; Rao and Pearse, 2016; Renthal et al., 2020; Shiers et al., 2020; Suzuki and Ochi, 2020; Lee and Cho, 2021). RAG genes encoding the activating protein 1 (AP-1) complex and the Sox (sex-determining region Y box) family were upregulated in both sexes. *Atf3* gene was upregulated 2.2-fold and 2.5-fold in both male and female DRG, respectively. This expression pattern consistently correlated with the increase of Atf3 and Atf3b protein isoforms (Figure 4A) encoded by the *Atf3* gene. *Sox11*-regulated *Sprr1a* gene encoding a small proline-rich protein (Jing et al., 2012) demonstrated more significant upregulation in females. In contrast, brain-derived neurotrophic factor (*Bdnf*), a stimulator of sciatic nerve axon growth (Zhou and Shine, 2003), was expressed higher in males than in females. Other male-prevalent RAGs included the AP-1 component c-Fos (*Fos*). RAGs were consistently upregulated at later stages of PNI, including sciatic nerve chronic constriction injury (CCI; Stephens et al., 2019; Ahlström et al., 2021). We suggest that their early activation and sustained long-term expression are indispensable for a robust response to injury.

#### Ion Channels, Neuroreceptors, and Transporters

The reduced expression of voltage-gated ion channels mRNA abundance persisted in both male and female DRG; however, the magnitude of the decrease was more significant in males. Subunits of voltage-dependent calcium channel subunits *Cacna2d2, Cacng2, and Cacng5* were downregulated. mRNA levels of voltage-gated sodium channels *Scn1a* (Nav1.1), *Scn4a* (Nav1.4), and *Scn5a* (Nav1.5), including DRG-specific channels *Scn8a* (Nav1.6), have decreased, except female-specific upregulation of epithelial sodium channel (*Scnn1b*). Potassium channel genes *Kcnj3, Kcnj4, and Kcng3* were downregulated. Notably, *Kcnk16* and *Kcnh4* were upregulated in both sexes, whereas *Kcnj13, Kcnq1*, and *Kcne3* were upregulated only in males. Ionotropic glutamate receptors AMPA (*Gria2*) was downregulated higher in males. We observed a male-specific increase of the immune cells-associated cannabinoid receptor (CBR) *Cnr2* mRNA.

#### Extracellular Matrix Homeostasis

**FIGURE 5:**
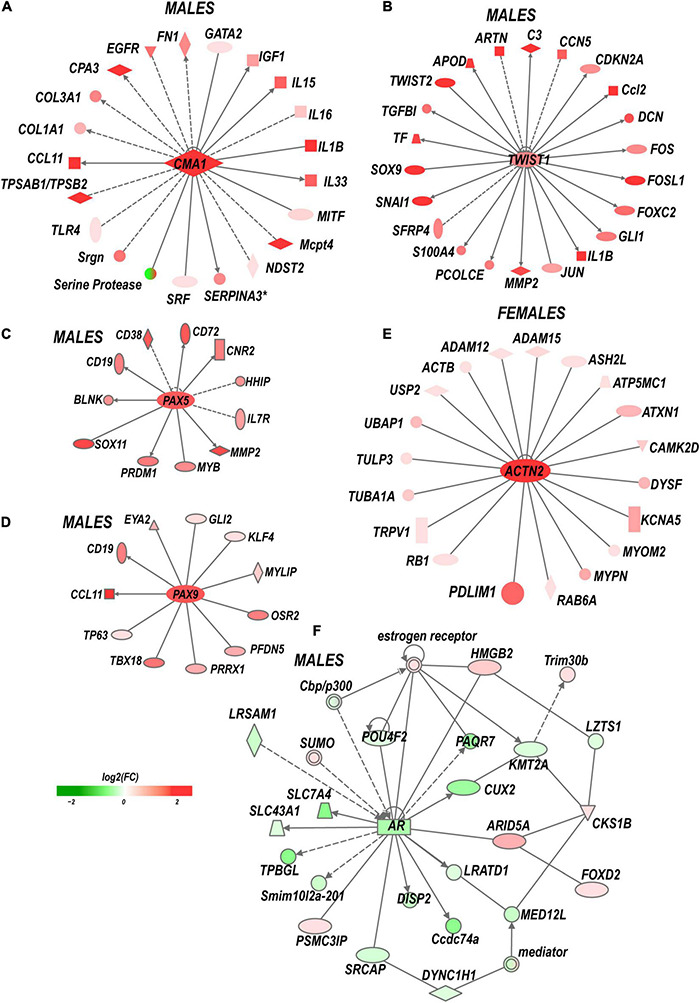
Sex-specific co-activation networks predicted by IPA. **(A)** Chimase (Cma1) network in males. **(B)** Twist1 network in males. **(C)** *Pax5* and **(D)** *Pax9* networks in males. **(E)** Actinin 2 (Actn2) network in females. **(F)** Co-downregulation of androgen receptor network in males. Gray arrow lines indicate the directionality of regulatory signaling. The intensity of red and green colors indicates up- and downregulation corresponding to Log_2_FC. Solid and dashed lines indicate direct and indirect interactions, respectively.

Males exhibited a more significant number of the upregulated ECM genes relative to females. *Mmp2, Mmp19*, *Adamts4, Adam10, and Adam17* were upregulated in males but did not change in females. *Mmp16, Adam8*, and *Adamts1* increased in both sexes. *Adamts16* decreased in males. The tissue inhibitor of metalloproteinases Timp1 gene, a stoichiometric activity regulator, was induced in both sexes, albeit the increase was higher in males. The *Timp1* expression pattern correlated with the increase of Timp1 protein in male DRG. Importantly, a higher absolute level of Timp1 mRNA and protein expression was detected in female sham DRG as compared to males (Figure 4B). In addition, we observed sexually dimorphic regulation of genes encoding the ECM structural molecules, including collagen and fibronectin species. TNF-inducible pentraxin-related protein *Ptx3* gene was induced in males. Ankyrin repeat protein *Ankrd2* upregulated in females and *Ankrd1* upregulated in both sexes. We uncovered an interactive protein network of serine proteases, including the extracellular chymotryptic serine protease *Cma1* (Figure 5A). According to the Ingenuity Pathway Analysis (IPA), the *Cma1* network can coordinate male-specific signaling receptor binding (*P* = 3.17e-5), integrins binding (*P* = 1.2e-5), cytokine activity (*P* = 5.56e-5), and microglial cell/macrophage activation (*P* = 1.6e-3).

#### Immune Response

Crucial genes encoding pattern-recognition receptors (PRPs) and TLRs exhibited male-specific upregulation. Cell surface *Tlr2* and all X-linked endosomal TLRs (*Tlr7, Tlr8*, and *Tlr13*) were upregulated in males. *Tlr5* was downregulated in males. mRNA levels encoding proteins that constitute the *TLR Signaling* axis: TIR-domain containing *Myd88* and *Trif* (*Ticam1*), *Irak3/4* kinases, interferon regulatory factors (*Irf*)-*3/4/8/9*, and NFkB family members *Nfkb1* and *Nfkb2* increased moderately in males.

The upregulation of cytokine and chemokine genes was generally more significant in males, affecting interleukin ligands (*Il1b, Il6, and Il33*) and interleukin receptors (*Il1rl1, Il1rn, Il2rg, 1l17rb*, and others). Remarkably, only interleukins *Il6, Il1a/b*, and *Il17ra* receptor genes were upregulated in females, consistent with the female-specific activity of *Il17a* activity in macrophage-sensory neuronal crosstalk (Luo et al., 2021). *Ccl2* [chemokine (C–C motif) ligand 2] exhibited robust upregulation in males. *Ccl2* change in females was lower, suggesting that *Ccl2* is a male-specific transcript activated by PNI events. Similarly, other chemokines, including *Ccl5, Ccl6, Ccl8, Ccl9, Ccl11, Ccl17, and Ccl21a*, were predominantly upregulated in males. *Ccl7, Ccl12*, and *Ccl19* were upregulated in both sexes. The macrophage colony-stimulating factor 1 (*Csf1*) gene was upregulated in both sexes, whereas the *Csf1* receptor (*Csf1r*) gene expression moderately increased only in male DRG. Notably, multiple genes related to the adaptive and innate immune responses according to GO terms GO:0045087 and GO:0002250, respectively, exhibited sexually dimorphic regulation.

#### Lipid Metabolism

Lipid carrier apolipoproteins *Apol6*, *Apo7c*, and apolipoprotein B receptor (*Apobr*) decreased in female DRG. *ApoL6, Apo7c, Apobr*, and Apolipoprotein D (*Apod*) genes were upregulated in male DRG. *Ch25h* gene, encoding the cholesterol-25-hydroxylase enzyme, responsible for the biosynthesis of the LXR ligand oxysterol (25-hydroxycholesterol), increased only in females.

#### Energy Metabolism

Both female and males DRG exhibited increased expression of multiple components of the complexes I, II, IV, and V (ATP synthases). Females specifically upregulated cytochrome c oxidase of complex IV (*Cox6a2*).

Upstream regulators. *Upstream regulators* affect the expression of multiple downstream genes, thus representing sex-specific signaling hubs in early PNI response. Upstream regulators were predicted using IPA, as follows:

*(i) Transcription factors (TFs)*. Multiple sexually dimorphic TFs were identified in our RNA-seq dataset. *Sox7* displayed upregulation in female DRG. Male DRG exhibited moderate upregulation of *Sox8* and *Sox10*, fork-head box (*Fox a1/c1/c2/d1/d2/s1*), and interferon activated (*Ifi209/207*) TFs. In males, co-regulatory networks centers around basic helix-loop-helix TFs *Twist1*/*2* (Figure 5B). According to our IPA predictions, the activated Twist1/2 network can promote macrophages-mediated inflammatory response (*P* = 1.7e-7). Two upregulated networks involving members of the paired box (*Pax5 and Pax9*) TFs were predicted (Figures 5C,D). The top canonical pathways regulated by these networks include *B Cell Development* (*P* = 2.1e-4), *IL-7* (*P* = 7.0e-4) and *IL-17A* (*P* = 3.4e-2) *Signaling pathways*, *PI3K Signaling in B Lymphocytes* (*P* = 2.3e-3), *Role of JAK1/JAK3 in Cytokines signaling* (*P* = 5.5e-4), *Primary Immunodeficiency* (*P* = 2.6e-6), and *Sonic Hedgehog signaling* (*P* = 1.5e-2). *Smad9* and *Pax2* were downregulated in males.

*(ii) ncRNAs.* We identified over 2500 significantly regulated ncRNA in both sexes, including 260 ncRNAs that changed expression >1.5-fold. Notably, the imprinted trans-regulator *H19* and the precursor gene for hematopoietic cell-specific *Mir124* exhibited male-specific regulation. *Snhg1, Snhg4*, and *Snhg5* host ncRNAs, each comprising one or more small nucleolar RNAs (snoRNAs), increased predominantly in males. In females, *Sox5os3* (an antisense transcript of *Sox5* gene) and *Lexis1* (a lipid-responsive LXR-induced inhibitor of cholesterol synthesis) ncRNAs were upregulated to a higher degree. It is worth noting that many ncRNAs are poorly characterized to date; therefore, their engagement in PNI responses will require future reassessment.

*(iii) Kinases.* We observed male-specific upregulation of protein tyrosine kinases (PTK) encoded by *Fgr, Hck*, and *Itk* genes. The Syk kinase-interacting TYRO (protein tyrosine kinase)-binding protein encoded by the *Tyrobp* gene (also known as *Dap12*) was upregulated in males. Most serine/threonine kinases, including mitogen-activated protein kinases (MAPK), protein kinases A (PKA), protein kinases B (AKT), protein kinases C (PKC), cyclin-dependent kinases (CDK), and Ca^2+^/calmodulin-dependent protein kinases (CAMK), did not significantly change or downregulated in both sexes. CAMK encoded by *Camk1*, and cyclin-dependent kinase inhibitor p21^*Cip*1^ (*Cdkn1a*) increased in both sexes.

*(iv) X/Y chromosome-linked genes.* We identified a higher upregulation of X-linked genes in males than females. Over 330 PNI-specific DEGs were linked to the sex chromosomes. Thirteen X-linked genes upregulated in females (*Taf7l, Dusp9, Llph-ps2, Timp1, Gpr119, Il2rg*, and others). At least 44 genes, including *Timp1*, were upregulated in males. Y-linked *Gm21860* lncRNA and the protein-coding *Gm47283* genes exhibited an increase in males.

*(v) Epigenetic factors.* Stress-sensing Gadd45 genes *(Gadd45a and Gadd45a*) upregulated in both sexes. Epigenetic writers and readers of histone code were differentially regulated as well. In males, the putative histone-lysine N-methyltransferase *Prdm6* and a bromodomain protein *Baz1a* genes were upregulated.

*(vi) Sex hormone receptors.* In females, the level of estrogen receptor (ESR) *Esr2* moderately increased. Activation of nociceptive signaling *via* ESR signaling has been implicated in female-specific pain hypersensitivity (Khomula et al., 2017; Chernov et al., 2020). In addition, because of the ESR’s direct interaction with the Nrip1 nuclear receptor (Cavaillès et al., 1995), the axonal outgrowth in females is likely mediated by the estrogen/ESR axis *via* co-regulated *Nrip1*, *Pdlim1/7* (PDZ and LIM domain-containing proteins), alpha-actinin 2 (*Actn2*) genes, and other interacting molecules (Figure 5E). In male DRG, the level of the ESRs did not significantly increase, whereas the androgen receptor (*Ar*) gene was downregulated. IPA-predicted co-downregulated Ar-interacting factors included *Pou4F2*, progestin and adipoQ receptor *Paqr*7, neuron-specific TF Cux2, sterol-sensing domains containing *Disp2*, Coiled-coil domain-containing *Ccsd74a*, E3 ubiquitin-protein ligase *Lrsam1*, and others (Figure 5F).

### Gene Ontology Analysis Untangled Sex-Specific Strategies

**FIGURE 6:**
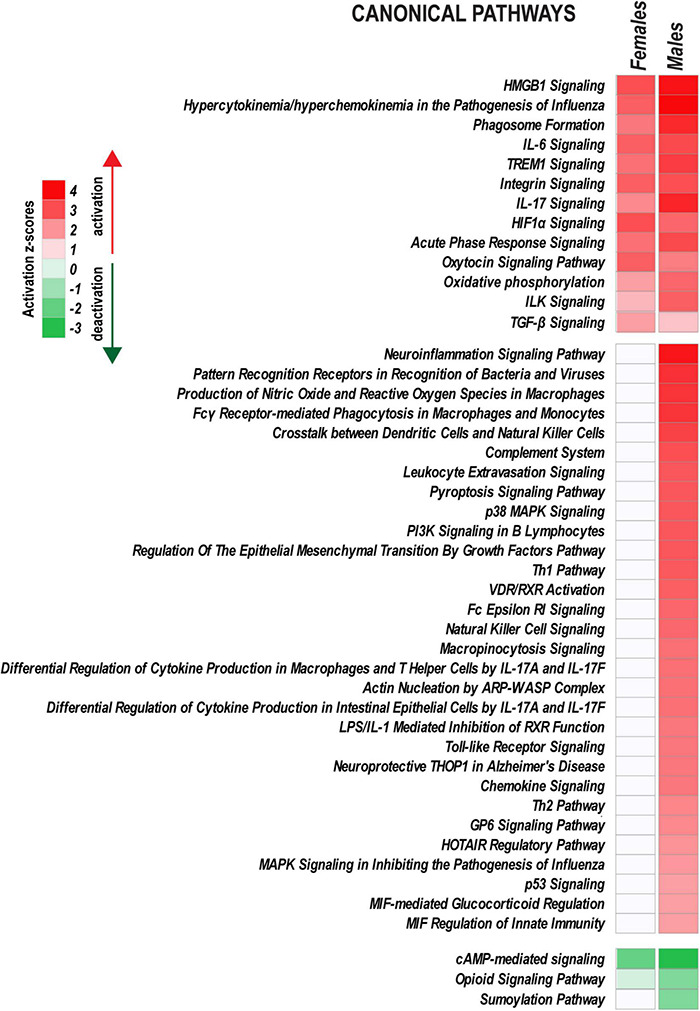
Ingenuity Pathway Analysis highlights sex-specific versus monomorphic early response mechanisms leveraged by PNI. Canonical pathways with |z-score| > 2.0 (identified by Fisher’s exact test) are shown on heatmaps. Positive and negative z-scores indicate upregulation (*z* > 2.0) or downregulation (*z* < –2.0) of respective pathways. Color intensity corresponds to higher (red) or lower (green) z-scores.

The prediction of relevant canonical signaling pathways induced in DRG at 24 h post-PNI (Figure 6) was conducted in IPA using a standard null hypothesis of the regulation randomness, a right-tailed Fisher’s exact test, and stringent statistical thresholds (padj < 0.1, | FC| > 2, activation | z-score| > 2).

Peripheral nerve injury activated a characteristic set of signaling pathways in both sexes, including *HMGB1 Signaling*, *TREM1 signaling*, *IL-6 Signaling*, *IL-17 Signaling, Integrin signaling*, *HIF1*α *Signaling*, *Oxytocin Signaling*, and *ILK Signaling*. Immunity responses (*Toll-like Receptor signaling, Chemokine signaling, Leukocyte extravasation signaling, Fc*γ *Receptor-mediated Phagocytosis in Macrophages* and others), *PI3K/AKT Signaling, HOTAIR regulatory pathway*, and *VDR/RXR Signaling* also increased in males. *cAMP-mediated signaling, Opioid Signaling, and Sumoylation pathways* were downregulated.

## Discussion

Recent transcriptomics data in animals identified sex-specific PNI responses in DRG (North et al., 2019; Ray et al., 2019; Stephens et al., 2019; Chernov et al., 2020; Mecklenburg et al., 2020; Paige et al., 2020; Tavares-Ferreira et al., 2020; Yu et al., 2020). However, sex differences in the immediate post-injury early-response transcriptional events remain not well understood. Herein, taking advantage of the sensitivity provided by high-depth RNA-seq analysis, we strived to untangle sexually dimorphic regulatory transcriptional mechanisms in murine DRG that develop at a 24 h time point after sciatic nerve axotomy. Other biological variables and experimental conditions, including species, strains, age, injury models, and timepoint, beyond the scope of this study, are expected to influence DRG transcriptomics.

*Nerve regenerative* program in damaged PNS afferent axons is orchestrated by neuron-specific RAG genes expressed in DRG (Bolin et al., 1995; Tsujino et al., 2000; Bosse et al., 2001; Snider et al., 2002; Abe and Cavalli, 2008; Sun and He, 2010; Van Kesteren et al., 2011; Ma and Willis, 2015; Chandran et al., 2016; Rao and Pearse, 2016; Renthal et al., 2020; Shiers et al., 2020; Suzuki and Ochi, 2020; Lee and Cho, 2021). In agreement, we observed upregulation of critical RAGs, including *Atf3, Sox11, Klf6, Sprr1a*, and *Gal.* Most transcriptional changes of RAGs were sexually monomorphic, except *Sprr1a* and galanin (*Gal*) that exhibited a higher increase in females as compared to males. Promoted by *Sox11*, *Sprr1a* is known to accelerate peripheral nerve regeneration (Jing et al., 2012). Cross-analysis with single-cell RNA-seq data in a spinal nerve injury model in mice (Renthal et al., 2020) indicated similar upregulation of *Atf3, Sox11, Sprr1a*, and other RAGs specific to injured neuron cells. At least two *Sox*-family genes exhibited upregulation. *Sox9* and *Sox11*. Sox proteins are evolutionarily conserved transcription factors sharing a similar HMG-box domain with the Y chromosome-linked Sry protein responsible for male sex determination. Sox proteins are architectural components of chromatin (Pevny and Lovell-Badge, 1997), which govern neuronal and glial lineage specification during development. In the regenerating adult PNS, Sox members are pioneer TFs that poise downstream TFs for transcriptional regulation (Stevanovic et al., 2021).

*Neuroinflammation* signaling pathway was induced within 24 h post-PNI in DRG of both sexes. However, males exhibited more robust upregulation of chemokines, stimulation of *Neuroinflammation Signaling*, and *Toll-like Receptor Signaling*. Males, but not females, exhibited robust DRG expression of at least 11 chemokine genes, including *Ccl2*, a known CCI-responding gene (Fu et al., 2010; LaCroix-Fralish et al., 2011) implicated in neuroinflammatory processes. Strong upregulation of *Tlr* genes in males included the X-linked endosomal *Tlr7, Tlr8*, and *Tlr13*, as well as moderate upregulation of *Irf(s), Myd88, Irak3/4*, and *Nfkb* genes. Remarkably, *Tlr5* decreased in males. *Tlrs* has been shown to mediate pain preferentially in males by activating cytokine and membrane lipid raft remodeling (Stokes et al., 2013; Ramachandran et al., 2019; Miller et al., 2020). The early-response activation of the *TLR Signaling* in male DRG can promote pro-inflammatory responses and drive chemokines and cytokines. *Il6*, expressed by neuronal, glial, and immune cells in male (Dubový et al., 2010) and female DRG (Ko et al., 2016), is induced at 24 h post-PNI. It is worth noting that neuronal *Il6* is also known as an axon growth-promoting RAG (Zhong et al., 1999; Cao et al., 2006; Ma and Willis, 2015). An important recent study demonstrated that IL17a directly activates nociceptor in mediating mechanical hypersensitivity selectively in female mice (Luo et al., 2021), consistent with the present finding of immediate and female-biased IL17ra induction in DRG.

Our data demonstrated monomorphic upregulation of injury-specific colony stimulation factor (Csf1) that is involved in macrophage proliferation and pain (Guan et al., 2016). However, the Csf1-receptor (*Csf1r*) and its downstream transmembrane adapter *Tyrobp* (*Dap12*) were upregulated in males. These findings may provide mechanistic insights into the mechanism of Csf1-induced macrophage expansion and pain regulation in male DRG reported on day 4 after spared nerve injury (Yu et al., 2020).

*Sensory function* is transcriptionally regulated by gene expression and axonal transport of chief neurotransmitter receptors, transporter, and ion channel genes. Their remarkable decline observed in both sexes is likely attributed to anterograde axonal transport to the regenerating nerve stump (Cavalli et al., 2005). The upregulated ion channels in both sexes, including *Kcnk16, Kcnh4*, and epithelial ENaC (*Scnn1b*), undergo axonal transport from DRG to peripheral terminals to participate in the transduction of painful sensation (García-Añoveros et al., 2001).

Under acute pathological conditions, estrogens can regulate a large spectrum of neuronal (Hucho et al., 2006) and immune functions (Straub, 2007) relevant to PNI. Estrogens (e.g., 17β-estradiol) activate nuclear *Esr1* and *Esr2* as well as membrane-bound GPCRs. While both female and male DRG display relatively high baseline *Esr1* and *Esr2* levels, the downstream signaling is likely influenced by the naturally higher 17β-estradiol levels in females. In the nervous system, rapid attenuation of estrogens in DRG can be complemented by neuron-specific biosynthesis (Evrard and Balthazart, 2004) in addition to estrogens of gonadal origin.

*Extracellular matrix regulation* in DRG affects markers of neurite growth, including ankyrin repeat protein *Ankrd2* (Tsukamoto et al., 2008) in females and *Ankrd1* (Stam et al., 2007) in both sexes. Male-specific upregulation of chimase (*Cma1*), extracellular *Mmp19*, *Adamts4*, and *Mmp2*, is coordinated with the robust expression of their stoichiometric regulator *Timp1*. Male-specific upregulation of the *Timp1* gene and higher baseline mRNA and protein levels in females relative to males strongly suggest distinct sexually dimorphic regulation of *Timp1* expression. We previously implicated epigenetic mechanisms in *Mmp*/*Timp* gene regulation (Chernov et al., 2009, 2010; Chernov and Strongin, 2011), resulting in rapid *Timp1* induction at the site of PNI and DRG (Kim et al., 2012; Chernov et al., 2015; Liu et al., 2015). Accordingly, we proposed that epigenetically controlled MMPs, ADAMTSs, and TIMPs remodel the ECM to promote axonal growth, cell differentiation, migration and survival, and neurovascular permeability to immune cells in a sex-specific manner (Shubayev and Strongin, 2018).

*Epigenetic cues* of sex-specific PNI response are robust in DRG. Previously considered as “junk” (Richard Boland, 2017), ncRNAs perform as potent epigenetic regulators of post-PNI remodeling (Yao and Yu, 2019; Li P. et al., 2021). A portion of ncRNA in DRG is trafficked by axonal transport (Cavalli et al., 2005) to the injury site to participate in regenerative processes. We identified over 740 regulated ncRNA in DRG transcriptomes in both sexes. The imprinted trans-regulator *H19* that modulates *PI3K Signaling* (Matouk et al., 2014) exhibited male-specific upregulation. Male-specific *Mir142hg*, the precursor gene for hematopoietic cell-specific *Mir124*, orchestrates innate immunity (Sharma, 2017; Shrestha et al., 2017) and neuropathic pain (Zhang et al., 2018; Li X. et al., 2021). *Snhg1, Snhg4*, and *Snhg5* host ncRNAs, each encoding one or more small nucleolar RNAs (snoRNAs), increased predominantly in males. In females, *Sox5os3* (an antisense transcript of the *Sox5* gene) was distinctly upregulated. We identified a novel marker of early PNI in both sexes, the *Lexis1* ncRNA, that may play a role in cholesterol metabolism and LXR pathway regulation (Sallam et al., 2016).

Upregulated Gadd45a/Gadd45g can guide epigenetic mechanisms responsible for *Atf3, c-Jun*, and *Bdnf* expression (Sultan and Sweatt, 2013). In addition, due to Gadd(s)/TET-dependent DNA demethylation, a broader gene activation may persist. Further studies of upstream regulators and DNA/histone methylation profiling are needed to establish sexually dimorphic characteristics of these epigenetic mechanisms in PNI.

*Genomic localization on X/Y chromosomes* likely contributes to sex-specific gene regulation in PNI. In contrast to males, in females, the RNA dosage is controlled by chromosome-wide epigenetic inactivation of one of the two X chromosomes (Xi), thus shutting off most Xi-linked transcripts. Because the X chromosome encodes the largest number of immunity-related genes (Bianchi et al., 2012), female immune cells rapidly leverage the expression of immune genes by partial reactivation of Xi (Syrett et al., 2017, 2019). We identified over 330 PNI-specific DEGs linked to the sex chromosomes, including female-specific upregulation of the X-linked TATA-box binding protein *Taf7l* (CT40) gene. The encoded Taf7l TF interacts with BORIS (a CTCF-family protein) (Rivero-Hinojosa et al., 2017) to promote long-range chromatin looping and regulation of large gene clusters (Zhou et al., 2014). These data imply a role of dynamic chromatin architecture in sexually dimorphic epigenetic regulation post-PNI. In males, transcripts on the Y chromosome, except for a short segment called the pseudoautosomal region (PAR), are not matched in the female genome. Two, albeit not well established, Y-linked genes were induced post-PNI in male DRG.

Additional considerations were given to the timing of sexually dimorphic changes, which directly relate to the temporal coordination of biological and behavioral processes. Generally, male DRG displayed a significantly higher number of differentially expressed genes, both up- and downregulated, in response to PNI within 24 h, than on day 7 (Ahlström et al., 2021) and day 14 PNI timepoints (Stephens et al., 2019). By comparing the most prominent sexually dimorphic transcripts at the early and later stages of PNI response, we observed consistent regulation of genes related to immunological response, neuronal transmission, regeneration, and plasticity obtained on day 7 (Ahlström et al., 2021) and day 14 PNI timepoints in rats (Stephens et al., 2019). However, as outlined in the present study, many sexually dimorphic and monomorphic transcripts were specific for the early stages of PNI response.

In conclusion, the present data provide insights into the regulation of the early-response transcriptional landscapes in DRG in female and male DRG after PNI. Epigenetic mechanisms emerged as essential hallmarks of sexually dimorphic regulation in early PNI response. The identity of the contributing cells in which these changes occur requires future studies to elucidate the functional activity of the respective genes and regulatory pathways in the context of specific cell types.

### Experimental Procedures

#### Reagents

Reagents and resources are listed in the resource table (Supplementary Table 2).

#### Animals

Female and male C57BL6/J mice (6–8 weeks old, Jackson Labs) were randomly assigned to axotomy (n = 6/group) and sham (*n* = 4/group). The mice were housed in a temperature-controlled room (∼22°C), on a 12-h light/dark cycle, and free access to food and water. All procedures were conducted between 8 and 12 daytime. Under isoflurane anesthesia, the left sciatic nerve was exposed at the mid-thigh level. The entire width of the nerve was axotomized using sterile microsurgery scissors. In a sham surgery animal cohort, nerves were exposed using surgical scissors without transection. The muscle was then sutured, and the skin stapled. At 24 h after surgery, lumbar L4 and L5 DRG were collected for RNA isolation. All animal procedures were performed according to the Policy on Humane Care and Use of Laboratory Animals and the protocol approved by the Institutional Animal Care and Use Committee at the VA San Diego Healthcare System.

#### Samples

All surgical and tissue harvesting instruments were sterilized and repeatedly treated with RNAse Away reagent followed by a rinse in RNAse-free water. Tissues were immediately submerged in 500 μl RNAlater Stabilization Solution to preserve RNA integrity, placed at 4°C overnight, and then transferred for storage at −20°C. All sample groups were processed in parallel to minimize batch effects.

#### RNA Purification

Dorsal root ganglia tissues from two animals were pooled to obtain at least 500 ng of total RNAs. Tissues were transferred in Trizol solution and disrupted by mechanical homogenization. Total RNAs were purified using RNeasy RNA purification reagents. RNA concentrations and quality were determined using Nanodrop absorbance ratios at 260/280 nm and 260/230 nm. RNA integrity was determined using the Agilent Bioanalyzer Nano RNA chip; 500 ng of total RNA samples (three replicates/group) with RIN ≥ 7.0 were used for RNA-seq.

#### RNA-seq

mRNA libraries were generated following the TruSeq Stranded mRNA library preparation protocol (Illumina). In brief, the poly-A enriched mRNAs were purified using poly-T oligo coupled magnetic beads, followed by mRNA fragmentation, first and second strand synthesis, cleaning on AMPure XP beads, and 3′-adenylation. Ligation of TruSeq dual-index adapters was used for barcoding. The quality of RNA-seq libraries was validated using qPCR. Libraries were sized on Agilent Bioanalyzer DNA high sensitivity chip and normalized. RNA-seq was performed using the paired-end 100 cycle program on the NovaSeq 6000 system at the Genomics High Throughput Facility (University of California Irvine). Base calls were recorded and converted to FASTQ files containing sequencing reads and the corresponding quality scores using Illumina software. Sequencing was conducted until we acquired at least 50 million paired-end reads per sample.

#### Data Alignment and Preparation for DEG Analysis

FASTQ files were uploaded to the Amazon S3 server and processed on Elastic Compute Cloud instances [EC2; Amazon Web Services (AWS)] running Ubuntu Server 20.04 LTS (64-bit ARM). Data analysis steps are summarized in Supplementary Figure 1 and Supplementary Table 2. FASTQ files were filtered to remove low-quality bases, TruSeq dual-index adapter sequences, and unpaired reads using Trimmomatic (Bolger et al., 2014). Transcript-level quantification was performed using Salmon (Patro et al., 2017) in quasi-mapping mode using version M27 of the Gencode mouse genome. To correct systematic biases commonly present in RNA-seq data, both *-seqBias* and *-gcBias* features were used. Transcript- to gene-level conversion was done using Tximeta (Love et al., 2020). RNA-seq quantification data quality was assessed using MultiQC (Ewels et al., 2016).

#### RNA-seq Data Analysis

Gene count matrices were imported into the DESeq2 package (Love et al., 2014). Outliers were identified by Cook’s distance method and excluded from further analysis. Dataset’s normalization was conducted using trimmed M-values (TMM) included in the DESeq2 package. Log2FC values were calculated using the Wald test. The adjusted (shrunken) log2FC values were calculated using the adaptive *t*-prior *apeglm* method (Zhu et al., 2019). Significant DEGs were identified by padj values below a false discovery rate (FDR) cutoff (padj < 0.1) (Supplementary Table 1). Padj < 0.1 were used in downstream analyses unless otherwise noted. Batch effects were controlled using removeBatchEffect (Ritchie et al., 2015) and RUVseq (Risso et al., 2014) functions. DEGs were visualized using ggVennDiagramm, PCAtools, ComplexHeatmap, and EnhancedVolcano R packages.

#### Signaling Pathway Analysis

Biological interpretation of the regulated signaling pathways in female and male animals was conducted by the Ingenuity Pathway Analysis (IPA) based on causal network approaches (Krämer et al., 2014). Cut-off filtering was applied (padj < 0.1, |Log2FC| > 0.58) for IPA. Signaling pathway regulation directionality was estimated based on statistical z-scores. Gene ontology terms were retrieved from Geneontology^1^.

#### Immunoblotting

DRG tissues were ground on ice in the lysis buffer (50 mM Tris-HCl, pH 7.6, 150 mM NaCl, 1% Triton X-100, 10% glycerol, 0.1% SDS, and 5 mM EDTA) supplemented with protease and phosphatase inhibitors (Thermo Fisher Scientific). Insoluble debris was removed by centrifugation (12,000 × *g*; 10 min). Protein concentration was measured using BCA protein assay. Lysates (15 μg of total protein) were separated by SDS-PAGE in 15% Tris-glycine gels (Bio-Rad). Proteins were transferred onto a PVDF membrane. The membrane was blocked in 5% non-fat milk (Bio-Rad) and incubated for 18 h at 4°C with monoclonal rabbit anti-Atf3 (1:1000 dilutions) or polyclonal goat anti-mouse Timp1 (1:1000 dilutions) followed by incubation for 1 h at ambient temperature with anti-rabbit IgG (1:2000 dilutions) or anti-goat (1:1000 dilutions) horseradish peroxidase-conjugated secondary antibody. For loading control, the membranes were re-probed using anti-GAPDH antibody (1:2000 dilutions). The blots were developed using SuperSignal West Dura Extended Duration Substrate kit (Thermo Fisher Scientific). Bands were digitized and quantitated using Chemidoc System (Bio-Rad).

#### Statistical Analyses

Digitized immunoblotting data were used to calculate means and standard deviation. Analysis of variance (ANOVA) with *post hoc* Tukey test was conducted in R (version 4.1.1)^2^ to determine statistical significance.

## Data Availability Statement

Publicly available datasets were analyzed in this study. This data can be found here: https://www.ncbi.nlm.nih.gov/geo/query/acc.cgi?acc=GSE182318.

## Ethics Statement

The animal study was reviewed and approved by the Institutional Animal Care and Use Committee at the VA San Diego Healthcare System.

## Author Contributions

AC: conceptualization, methodology, software, formal analysis, investigation, data curation, writing – original draft, writing – review and editing, and visualization. VS: conceptualization, resources, project administration, funding acquisition, writing – original draft, and review and editing. Both authors contributed to the article and approved the submitted version.

## Author Disclaimer

The content is solely the authors’ responsibility and does not necessarily represent the official views of the funding agencies.

## Conflict of Interest

The authors declare that the research was conducted in the absence of any commercial or financial relationships that could be construed as a potential conflict of interest.

## Publisher’s Note

All claims expressed in this article are solely those of the authors and do not necessarily represent those of their affiliated organizations, or those of the publisher, the editors and the reviewers. Any product that may be evaluated in this article, or claim that may be made by its manufacturer, is not guaranteed or endorsed by the publisher.

## Footnotes

1
http://geneontology.org

2
https://www.R-project.org

## Supplementary Material

Supplementary Figure 1 RNA-seq analysis workflow.

Supplementary Table 1 Post-axotomy (24 h) DEGs in murine female and male DRG.

Supplementary Table 2 Key resources table.

## Abbreviations

CNS: central nervous system
DEG: differentially expressed gene
DRG: dorsal root ganglia
FC: fold change
FDR: false discovery rate
IPA: Ingenuity Pathway Analysis
ncRNA: non-coding RNAs
padj: adjusted *p*-values
PNI: peripheral nerve injury
PNS: peripheral nervous system
RAG: regeneration-associated gene
RNA-seq: RNA sequencing
TF: transcription factor
TLR: Toll-like receptors.
