# Supplementary figures and images for: Sexual Dimorphism of Early Transcriptional Reprogramming in Dorsal Root Ganglia After Peripheral Nerve Injury
Source: Front Mol Neurosci. 2021 Dec 13;14:779024. doi: 10.3389/fnmol.2021.779024 (PMC8710713; doi:10.3389/fnmol.2021.779024)

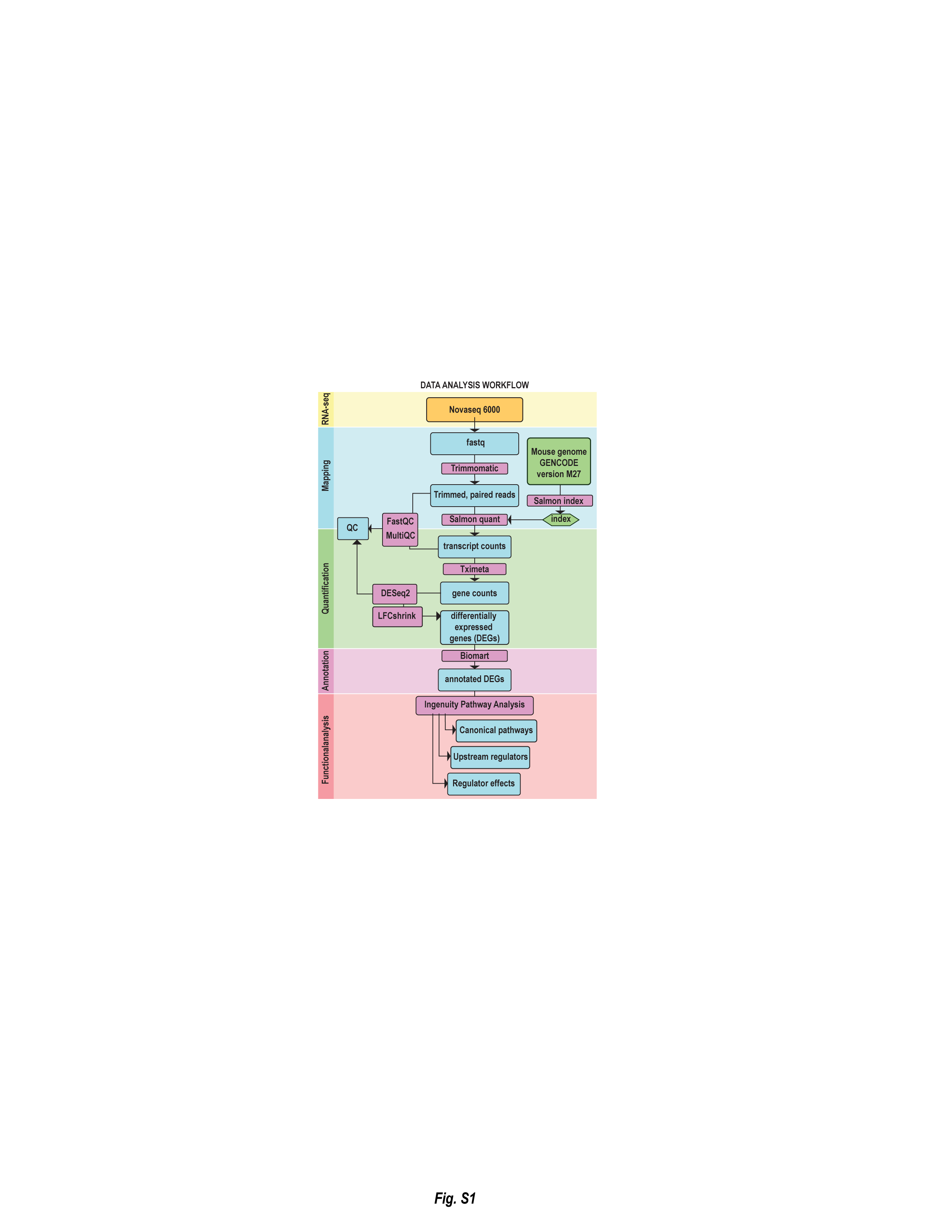

Supplement: Supplementary Figure 1 — RNA-seq analysis workflow. [file Image_1.TIF]
