# Supplementary material for: Sexual Dimorphism of Early Transcriptional Reprogramming in Dorsal Root Ganglia After Peripheral Nerve Injury
Source: Front Mol Neurosci. 2021 Dec 13;14:779024. doi: 10.3389/fnmol.2021.779024 (PMC8710713; doi:10.3389/fnmol.2021.779024)
Supplement: Supplementary Table 2 — Key resources table. [file Data_Sheet_2.PDF]

Table S2. Key resources table

Resource	Description	Source	Identifier	URL
**Animals**	Mice	Jackson Labs, Sacramento, CA USA	strain C57BL/6	
**Reagent**	Trizol reagent	Thermo Fisher Scientific, Carlsbad, CA USA	15596026	https://www.thermofisher.com/order/catalog/product/15596026
**Reagent**	RNAlater Stabilization Solution	Thermo Fisher Scientific, Carlsbad, CA USA	AM7020	https://www.thermofisher.com/order/catalog/product/AM7020
**Reagent**	RNAeasy Mini Kit	Qiagen, Carlsbad, CA	74104	
**Reagent**	Halt™ Protease and Phosphatase Inhibitor Cocktail, EDTA-free (100X)	Thermo Fisher Scientific, Carlsbad, CA USA	78441	https://www.thermofisher.com/order/catalog/product/78441
**Reagent**	Pierce™ BCA Protein Assay Kit	Thermo Fisher Scientific, Carlsbad, CA USA	23227	https://www.thermofisher.com/order/catalog/product/23227
**Reagent**	SuperSignal™ West Dura Extended Duration Substrate	Thermo Fisher Scientific, Carlsbad, CA USA	34076	https://www.thermofisher.com/order/catalog/product/34076
**Antibody**	ATF-3 (E9J4N) Rabbit mAb	Cell Signaling Technology, Danvers, MA USA	18665	https://www.cellsignal.com/products/primary-antibodies/atf-3-e9j4n-rabbit-mab/18665
**Antibody**	Goat polyclonal Mouse TIMP-1 IgG Antibody	R&D Systems, Minneapolis, MN USA	AF980	https://www.rndsystems.com/products/mouse-timp-1-antibody_af980
**Antibody**	Anti-rabbit IgG, HRP-linked Antibody	Cell Signaling Technology, Danvers, MA USA	7074	https://www.cellsignal.com/products/secondary-antibodies/anti-rabbit-igg-hrp-linked-antibody/7074
**Antibody**	Goat IgG HRP-conjugated Antibody	R&D Systems, Minneapolis, MN USA	HAF017	https://www.rndsystems.com/products/goat-igg-hrp-conjugated-antibody_haf017
**Antibody**	Anti-GAPDH Antibody (6C5)	Santa Cruz Biotechnology, Dallas, TX USA	sc-32233	https://www.scbt.com/p/gapdh-antibody-6c5
**Equipment**	Centrifuge	Eppendorf	5425	
**Equipment**	Tapestation	Agilent Technologies, San Diego, CA USA	4200	
**Equipment**	Novaseq	Illumina Inc., San Diego, CA USA	6000	

**Software**	Trimmomatic	(1)	Version 0.39	https://www.usadellab.org/cms/?page=trimmomatic
**Software**	Salmon	(2)	Version 1.3.0	https://salmon.readthedocs.io
**Software**	Tximeta	(3)	Version 3.11	https://www.bioconductor.org/packages/release/bioc/html/tximeta.html
**Software**	MultiQC	Stockholm University, Stockholm, Sweden (4)	Version 1.9	https://multiqc.info/docs/
**Software**	DESeq2	(5)	Version 1.33.4	https://www.bioconductor.org/packages/devel/bioc/vignettes/DESeq2/inst/doc/DESeq2.html
**Software**	Ingenuity Pathway Analysis	Qiagen, Carlsbad, CA	Version Fall 2021	https://digitalinsights.qiagen.com
**Software**	PCAtools	Kevin Blighe	Version 2.4	https://github.com/kevinblighe/PCAtools
**Software**	Complex Heatmap	Zuguang Gu (6)	Version 2.8	
**Software**	Enhanced Volcano	Kevin Blighe	Version 1.10.0	https://github.com/kevinblighe/EnhancedVolcano
**Database**	Geneontology		09.01.2021	http://geneontology.org
**Database**	GENCODE	(7)	Version M27	https://www.gencodegenes.org/mouse/

References

1. Bolger AM, Lohse M, Usadel B. Trimmomatic: a flexible trimmer for Illumina sequence data. *Bioinformatics* (2014) 30(15):2114-20. Epub 2014/04/04. doi: 10.1093/bioinformatics/btu170. PubMed PMID: 24695404; PubMed Central PMCID: PMCPMC4103590.
2. Patro R, Duggal G, Love MI, Irizarry RA, Kingsford C. Salmon provides fast and bias-aware quantification of transcript expression. *Nat Methods* (2017) 14(4):417-9. doi: 10.1038/nmeth.4197. PubMed PMID: 28263959; PubMed Central PMCID: PMCPMC5600148.
3. Love MI, Soneson C, Hickey PF, Johnson LK, Pierce NT, Shepherd L, et al. Tximeta: Reference sequence checksums for provenance identification in RNA-seq. *PLoS Comput Biol* (2020) 16(2):e1007664. Epub 2020/02/26. doi: 10.1371/journal.pcbi.1007664. PubMed PMID: 32097405; PubMed Central PMCID: PMCPMC7059966 following competing interests: RP is a co-founder of Ocean Genomics.
4. Ewels P, Magnusson M, Lundin S, Käller M. MultiQC: summarize analysis results for multiple tools and samples in a single report. *Bioinformatics* (2016) 32(19):3047-8. Epub 2016/06/18. doi: 10.1093/bioinformatics/btw354. PubMed PMID: 27312411; PubMed Central PMCID: PMCPMC5039924.
5. Love MI, Huber W, Anders S. Moderated estimation of fold change and dispersion for RNA-seq data with DESeq2. *Genome Biol* (2014) 15(12):550. Epub 2014/12/18. doi: 10.1186/s13059-014-0550-8. PubMed PMID: 25516281; PubMed Central PMCID: PMCPMC4302049.
6. Gu Z, Eils R, Schlesner M. Complex heatmaps reveal patterns and correlations in multidimensional genomic data. *Bioinformatics* (2016) 32(18):2847-9. doi: 10.1093/bioinformatics/btw313.

7. Frankish A, Diekhans M, Ferreira AM, Johnson R, Jungreis I, Loveland J, et al. GENCODE reference annotation for the human and mouse genomes. *Nucleic Acids Res* (2019) 47(D1):D766-d73. Epub 2018/10/26. doi: 10.1093/nar/gky955. PubMed PMID: 30357393; PubMed Central PMCID: PMCPMC6323946.
